# A20 Inhibits LPS-Induced Inflammation by Regulating TRAF6 Polyubiquitination in Rainbow Trout

**DOI:** 10.3390/ijms22189801

**Published:** 2021-09-10

**Authors:** Ju Hye Jang, Hyun Kim, In Young Jung, Ju Hyun Cho

**Affiliations:** 1 Research Institute of Life Sciences, Gyeongsang National University, Jinju 52828, Korea; juhye.jang@gnu.ac.kr (J.H.J.); hyun.kim@gnu.ac.kr (H.K.); 2 Division of Applied Life Science (BK21Four), Gyeongsang National University, Jinju 52828, Korea; Inyoung.jung@gnu.ac.kr; 3 Division of Life Science, Gyeongsang National University, Jinju 52828, Korea

**Keywords:** A20 (TNFAIP3), TRAF6, deubiquitinase (DUB), rainbow trout, RTH-149

## Abstract

The ubiquitin-editing enzyme A20 is known to inhibit the NF-κB transcription factor in the Toll-like receptor (TLR) pathways, thereby negatively regulating inflammation. However, its role in the TLR signaling pathway in fish is still largely unknown. Here, we identified a gene encoding A20 (OmA20) in rainbow trout, *Oncorhynchus mykiss*, and investigated its role in TLR response regulation. The deduced amino acid sequence of OmA20 contained a conserved N-terminal ovarian tumor (OTU) domain and seven C-terminal zinc-finger (ZnF) domains. Lipopolysaccharide (LPS) stimulation increased OmA20 expression in RTH-149 cells. In LPS-stimulated RTH-149 cells, gain- and loss-of-function experiments revealed that OmA20 inhibited MAPK and NF-κB activation, as well as the expression of pro-inflammatory cytokines. OmA20 interacted with TRAF6, a key molecule involved in the activation of TLR-mediated NF-κB signaling pathways. LPS treatment increased the K63-linked polyubiquitination of TRAF6 in RTH-149 cells, which was suppressed when OmA20 was forced expression. Furthermore, mutations in the OTU domain significantly decreased deubiquitination of the K63-linked ubiquitin chain on TRAF6, indicating that deubiquitinase activity is dependent on the OTU domain. These findings suggest that OmA20, like those of mammals, reduces LPS-induced inflammation in rainbow trout, most likely by regulating K63-linked ubiquitination of TRAF6.

## 1. Introduction

The innate immune system, an evolutionarily conserved component of the host defense, provides initial protection against infectious pathogens [1,2]. It relies on various germline-encoded pattern recognition receptors (PRRs) expressed on the cell surface, in intracellular compartments, or secreted into the bloodstream and tissue fluids to recognize pathogen-associated molecular patterns (PAMPs), which are evolutionarily conserved structures on pathogens [3,4]. The major PRRs include Toll-like receptors (TLRs), Nod-like receptors (NLRs), retinoic acid-inducible gene I (RIG-I)-like receptors (RLRs), and C-type lectin receptors (CLRs) [4].

Among the various PRRs, the TLR family is one of the most well-studied PRR families responsible for detecting invading pathogens both outside and inside the cell [5]. Upon recognizing respective PAMPs, TLRs recruit an adaptor molecule, myeloid differentiation factor 88 (MyD88). TLR-MyD88 interaction recruits and activates interleukin receptor-associated kinase (IRAK) 1 and 4. When IRAKs are activated, they interact with tumor necrosis factor receptor-associated factor 6 (TRAF6), an E3 ligase, causing TRAF6 to be auto-ubiquitinated on lysine 63 (K63) [6]. Ubiquitinated TRAF6 on K63 serves as a recognition signal for the recruitment of transforming growth factor-β-activated kinase 1 (TAK1) binding protein (TAB) 2/3, which activates TAK1. Following TAK1 activation, NF-κB and mitogen-activated protein kinase (MAPK) are activated, resulting in the production of inflammatory cytokines [7,8]. Although TLR signaling and pro-inflammatory cytokine release are required to eliminate invading pathogens, excessive activation can lead to autoimmune and inflammatory diseases [9]. As a result, TLR signaling must be strictly regulated to avoid an overwhelming inflammatory response.

Several mechanisms are responsible for the regulation of the TLR signaling pathways. These include physical interactions, conformational changes, phosphorylation, ubiquitination, and proteasome-mediated degradation of numerous regulatory proteins [10,11,12]. Among the various mechanisms, ubiquitination, the covalent attachment of ubiquitin to a lysine residue inside the substrate protein, is a post-translational modification that regulates the modified protein’s stability, function, and localization [13,14]. A cascade of enzymes, including ubiquitin-activating enzymes (E1), ubiquitin-conjugating enzymes (E2), and ubiquitin ligases (E3), mediates the transfer of ubiquitin molecules onto the targeted protein substrate [15,16]. The ubiquitination process is counteracted by deubiquitinases (DUBs), specialized proteases removing ubiquitin from the substrate [17]. Recent studies have shown that A20, encoded by the tumor necrosis factor-α (TNF-α)-induced protein 3 gene (TNFAIP3), plays a vital role in strictly controlling inflammation in mammals by negatively regulating the TLR signaling pathway [18,19,20]. A20 is a ubiquitin-editing enzyme that functions as both an E3 ligase and a DUB. It also has specialized ubiquitin-binding domains (ZnF domains) that control innate immune signaling pathways [21,22,23,24]. In mammals, A20 inactivates TRAF6 and receptor-interacting protein 1 (RIP1) by cleaving K63-linked polyubiquitin and promoting K48-linked polyubiquitination that triggers proteasome-mediated degradation [25,26,27], while A20 silencing promotes NF-κB/TRAF6 activation [28].

Orthologs of human A20 have been identified in several fish species, including zebrafish (*Danio rerio*) [29], fathead minnow (*Pimephales promelas*) [30], and Japanese pufferfish (*Takifugu rubripes*) [31]. Fish A20s, such as those of mammals, might play a key role in maintaining inflammatory homeostasis. Indeed, it has been recently reported that A20-deficient zebrafish are hyper-responsive to microbial immune activation and die prematurely [32]. The function of fish A20s, on the other hand, is little understood other than the fact that some fish A20s are involved in the nucleotide-binding oligomerization domain-containing protein (NOD) signaling pathways. Oehlers et al. investigated the NOD signaling pathway in zebrafish using in situ hybridization and quantitative real-time PCR (qRT-PCR) techniques and detected A20 in larvae, particularly in the intestine and kidney, as well as in leucocytes [29]. In a previous study, we showed that peptidoglycan recognition protein (PGRP) controls the activation of the NOD-mediated NF-κB pathway through A20 expression in rainbow trout [33]. Other signaling pathways might be affected by A20s in fish. For example, Merour et al. reported that fathead minnow A20, which is upregulated by the RIG-I pathway, acts as a negative feedback regulator of RIG-I-mediated antiviral induction by interrupting RIG-I signaling at the level of TANK-binding kinase 1 (TBK1), a critical kinase involved in interferon expression [30]. On the other hand, little is known about the function of fish A20 in TLR signaling pathways. In this study, we identified and characterized A20 (OmA20) in rainbow trout, *Oncorhynchus mykiss*, one of the most significant cold-water fish species because it is essential for food production and sport fishing, as well as a research model. In particular, we investigated the role of OmA20 in the regulation of TLR responses in a rainbow trout hepatoma cell line RTH-149.

## 2. Results

### 2.1. Sequence and Characteristics of OmA20

**Figure 1:**
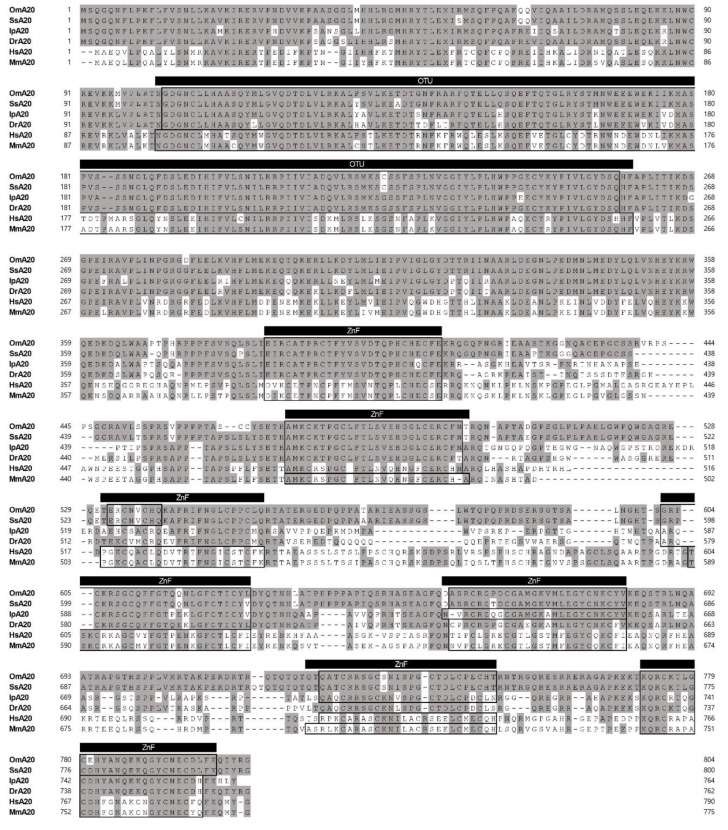
Multiple alignments of deduced amino acid sequences of rainbow trout A20 (*Oncorhynchus mykiss*, Om) with those of Atlantic salmon (*Salmo salar*, Ss), channel catfish (*Ictalurus punctatus*, Ip), zebrafish (*Danio rerio*, Dr), human (*Homo sapiens*, Hs), and mouse (*Mus musculus*, Mm). Identical amino acids are shaded grey. OTU and ZnF domains predicted by SMART program are indicated as black bars above the alignment.

**Figure 2:**
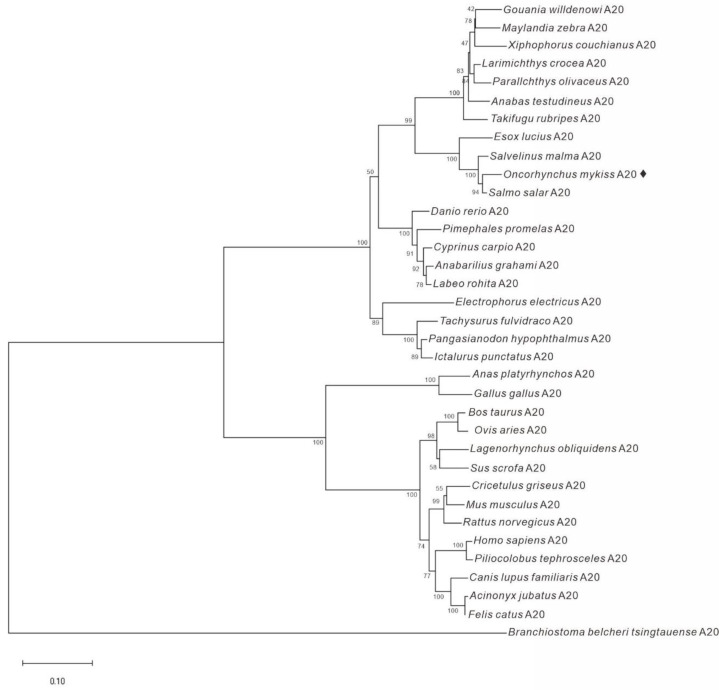
Phylogenetic tree analysis of A20 protein from various species. A phylogenetic tree analysis was carried out using the Neighbor-Joining method with 10,000 bootstrap replicates in the MEGA-X software.

We identified an A20 candidate (OmA20) from the total RNA of rainbow trout using 5′- and 3′-RACE. The cDNA of OmA20 (GenBank Accession number MF671983) is 3367 bp long and contains an open reading frame (ORF) of 2415 bp, a 5′-untranslated region (UTR) of 162 bp, and a 3′-UTR of 790 bp. The ORF encodes a deduced protein of 804 amino acids with an estimated molecular mass of 90.2 kDa. Secondary structure prediction by SMART revealed that OmA20 contained an N-terminal ovarian tumor (OTU) domain (103–257 aa) and seven C-terminal zinc-finger (ZnF) domains (386–411, 473–498, 532–539, 602–627, 657–682, 726–750, and 772–799 aa) (Figure S1). The whole-genome sequencing of rainbow trout recently revealed a homologous gene that encodes two TNFAIP3 isoforms. A comparison of TNFAIP3 domain architectures showed that the two isoforms had similar domain organization to that of OmA20. Among the two isoforms, the amino acid sequence of TNFAIP3 isoform X2 (GenBank Accession number XP_036812005.1) was identical to that of OmA20. The alignment of OmA20 with A20s from Atlantic salmon (*Salmo salar*), channel catfish (*Ictalurus punctatus*), zebrafish (*D. rerio*), human (*Homo sapiens*), and mouse (*Mus musculus*) revealed that OmA20 contains a highly conserved OTU domain, which is required for DUB activity (Figure 1). A phylogenetic tree was constructed using full-length A20 protein from a variety of species to understand OmA20′s evolutionary status (Figure 2). The result revealed that these homolog proteins could be divided into three groups: teleost, tetrapod (birds and mammals), and lancelet. Among the teleost species, the OmA20 protein formed a sub-cluster with two other Salmonidae family members, *Salvelinus malma* and *S. salar*, and one Esocidae family member, *Esox lucius* (closest to *S. salar*).

### 2.2. OmA20 Expression in Fish Tissues and RTH-149 Cells Stimulated with LPS

**Figure 3:**
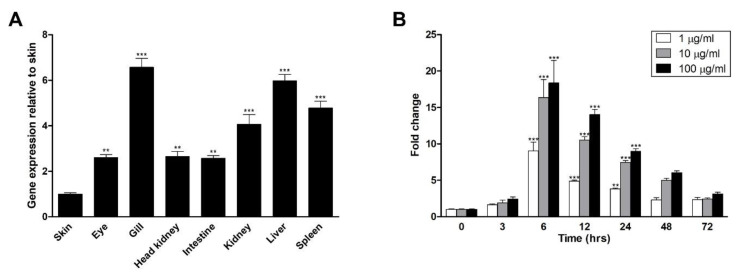
OmA20 expression in fish tissues (**A**) and RTH-149 cells stimulated with LPS (**B**). (**A**) The relative level of OmA20 mRNA expression was evaluated in tissues of rainbow trout using qRT-PCR. Results from qRT-PCR were expressed as a relative fold change compared to skin after normalization against EF1-α. (**B**) RTH-149 cells were stimulated with LPS at different doses (1, 10, and 100 μg/mL), and the expression of OmA20 was measured by qRT-PCR at indicated time points. OmA20 expression level was normalized by the EF1-α level and shown as fold change compared with control cells that had not been stimulated as a value of 1. ** *p* < 0.01, *** *p* < 0.001.

The tissue-specific expression of OmA20 was examined to select a suitable cell line for in vitro studies. As shown in Figure 3A, OmA20 was expressed in all tissues tested, despite variances in transcript levels. Based on the tissue-specific expression pattern data, we chose RTH-149, a cell line derived from liver tissue, which showed a relatively high level of OmA20 expression. Then, to test the effect of the TLR ligand lipopolysaccharide (LPS) on OmA20 expression, we stimulated RTH-149 cells with LPS. LPS increased OmA20 expression in RTH-149 cells in a dose-dependent manner, with the highest level at 6 h after stimulation (Figure 3B).

### 2.3. Involvement of OmA20 on MAPK and NF-κB Activation in RTH-149 Cells Stimulated with LPS

**Figure 4:**
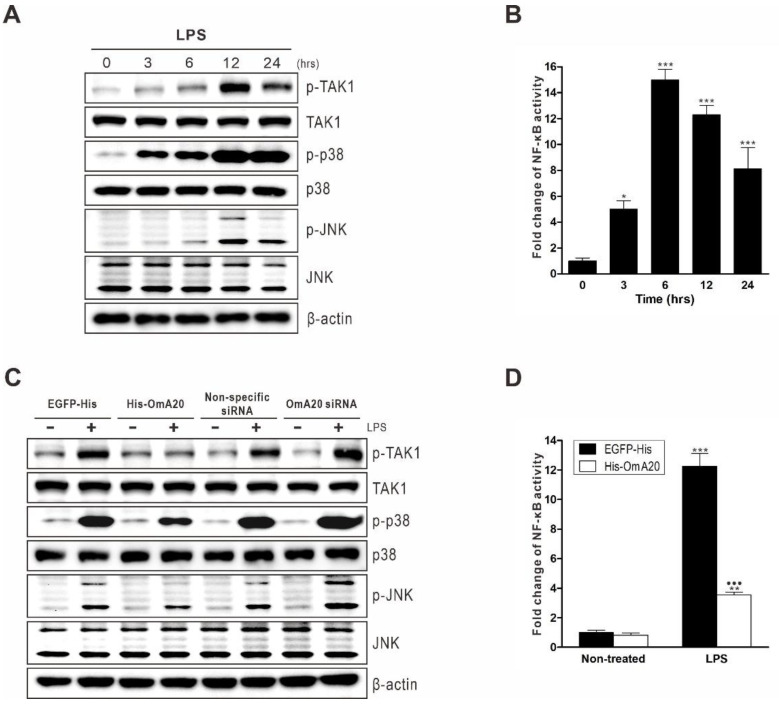
Involvement of OmA20 on MAPK and NF-κB activation in RTH-149 cells stimulated with LPS. (**A**) After stimulating RTH-149 cells with LPS (10 μg/mL) for the indicated time periods, phosphorylated, and total levels of TAK1, p38 MAPK, and JNK were determined by western blot analysis. (**B**) RTH-149 cells, co-transfected with pNF-κB-Luc and pRL-TK for 48 h, were stimulated with LPS (10 μg/mL) for the indicated time periods. Then, NF-κB activity was determined using a dual-luciferase reporter assay system. (**C**) RTH-149 cells, transfected with His-OmA20, EGFP-His, OmA20 siRNA, or non-specific siRNA for 48 h, were stimulated with LPS (10 μg/mL) for 12 h. Then, phosphorylated and total levels of TAK1, p38 MAPK, and JNK were determined by western blot analysis. (**D**) RTH-149 cells, co-transfected with a His-OmA20 or EGFP-His along with pNF-κB-Luc and pRL-TK for 48 h, were stimulated with LPS (10 μg/mL) for 12 h. Then, NF-κB activity was determined using a dual-luciferase reporter assay system. * *p* < 0.05, ** *p* < 0.01, *** *p* < 0.001 unstimulated control vs. LPS-stimulated cells; ^●●●^ *p* < 0.001 EGFP-His vs. His-OmA20.

TLR coding sequences and functions are substantially conserved in vertebrates. Similarly, TLR-mediated signaling pathways are highly conserved [34,35]. In mammals, the binding of LPS to the TLR4 receptor complex initiates the recruitment of several adaptor proteins. It triggers the activation of various signaling pathways, which eventually leads to the activation of MAPKs and NF-κB, resulting in the expression of pro-inflammatory genes [36]. When RTH-149 cells were stimulated with LPS, the phosphorylation of TAK1, p38 MAPK, and jun-amino-terminal kinase (JNK) was increased 12 h later in the same way as those in mammals [37] (Figure 4A). Furthermore, at 6 h after LPS stimulation, NF-κB activity was raised 14.98-fold relative to control cells that had not been activated (Figure 4B). In mammals, A20 has been known to negatively regulate TLR signaling by interfering with NF-κB and MAPK signaling pathways [38]. Therefore, we performed gain- and loss-of-function experiments to see if OmA20 is involved in activating TAK1, p38 MAPK, and JNK in RTH-149 cells stimulated with LPS. Cells were first transfected with His-OmA20 or siRNA targeting OmA20 to overexpress or silence OmA20, respectively (Figure S2). OmA20 overexpression significantly reduced the phosphorylation of TAK1, p38 MAPK, and JNK in RTH-149 cells 12 h after LPS stimulation, whereas OmA20 silencing increased phosphorylation (Figure 4C). OmA20 overexpression also decreased NF-κB activity in LPS-stimulated RTH-149 cells by ~71% (Figure 4D). When RTH-149 cells transfected with enhanced green fluorescent protein (EGFP)-His were stimulated with LPS for 12 h, NF-κB activity increased 12.24-fold compared to control cells that had not been stimulated. OmA20 overexpression, on the other hand, decreased NF-κB activity in LPS-stimulated cells by 3.55-fold.

**Figure 5:**
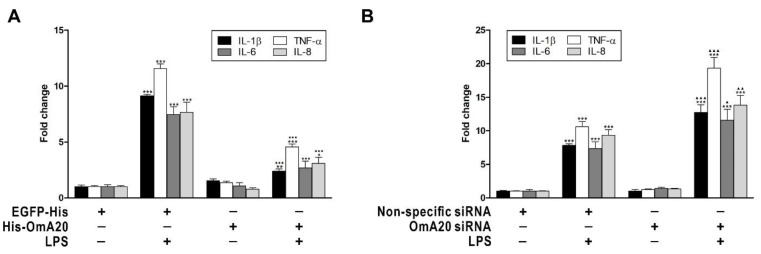
Effect of OmA20 overexpression or silencing on the expression of downstream target genes of MAPKs and NF-κB in RTH-149 cells stimulated with LPS. RTH-149 cells, transfected with His-OmA20, EGFP-His (**A**), OmA20 siRNA, or non-specific siRNA (**B**) for 48 h, were stimulated with LPS (10 μg/mL) for 12 h. Then, the expression of IL-1β, TNF-α, IL-6, and IL-8 was analyzed by qRT-PCR. * p < 0.05, ** p < 0.01, *** p < 0.001 unstimulated control vs. LPS-stimulated cells; ^●●●^ *p* < 0.001 EGFP-His vs. His-OmA20; ^▲^ *p* < 0.05, ^▲▲^ *p* < 0.01, ^▲▲▲^ *p* < 0.001 non-specific siRNA vs. OmA20 siRNA.

Next, we investigated the effect of OmA20 overexpression or silencing on the expression of downstream target genes of MAPKs and NF-κB, such as IL-1β, TNF-α, IL-6, and IL-8 in RTH-149 cells stimulated with LPS. At 12 h after LPS stimulation, the expression of IL-1β, TNF-α, IL-6, and IL-8 in RTH-149 cells transfected with EGFP-His (or non-specific siRNA) was elevated by 9.13- (7.84-), 11.57- (10.6-), 7.45- (7.34-), and 7.64-fold (9.35-fold), respectively, compared to control cells that had not been activated (Figure 5). OmA20 overexpression decreased the expression of IL-1β (2.39-fold), TNF-α (4.56-fold), IL-6 (2.69-fold), and IL-8 (3.08-fold) in RTH-149 cells 12 h after LPS stimulation (Figure 5A), whereas OmA20 silencing substantially increased the expression of IL-1β (12.72-fold), TNF-α (19.33-fold), IL-6 (11.56-fold), and IL-8 (13.85-fold) (Figure 5B). Overall, these findings imply that A20 may act as a negative regulator of TLR signaling in rainbow trout in the same way as it does in mammals.

### 2.4. Effect of OmA20 on the Ubiquitination of TRAF6 in LPS-Stimulated RTH-149 Cells

**Figure 6:**
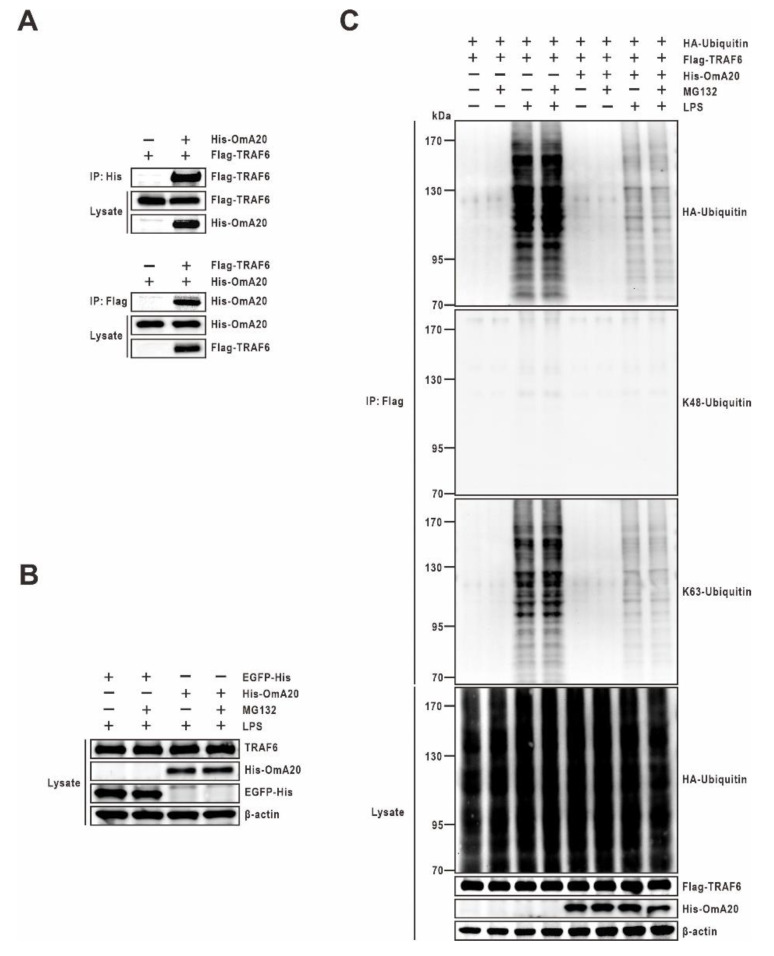
Effect of OmA20 overexpression on the ubiquitination of TRAF6 in RTH-149 cells stimulated with LPS. RTH-149 cells were transfected with expression vectors for various combinations (above lanes) of His-OmA20, Flag-TRAF6, EGFP-His, and HA-Ubiquitin. (**A**) After transfection, cells were stimulated with LPS (10 μg/mL) for 12 h. The His-OmA20 or Flag-TRAF6 complexes were isolated by immunoprecipitation (IP) followed by immunoblot (IB) to detect the interaction between OmA20 and TRAF6. Cell lysates were subjected to IB to detect the indicated proteins. (**B**,**C**) After transfection, cells (except control cells) were pretreated with 10 μg/mL LPS for 12 h and treated with or without MG132 (25 μM) for an additional 4 h. Cell lysates were subjected to IB to detect the indicated proteins. (**C**) Flag-TRAF6 was isolated by IP followed by IB (with anti-HA, anti-K48-linkage specific polyubiquitin, and anti-K63-linkage specific polyubiquitin antibodies) to examine its ubiquitin conjugation. All data are representative of three or more experiments.

TRAF6 is a key transduction molecule downstream of the TLR pathways whose polyubiquitination can induce NF-κB activity and the subsequent secretion of inflammatory factors [39,40]. In mammals, A20 inhibits the polyubiquitination and activation of the E3 ubiquitin ligase TRAF6 in the TLR pathways [18]. However, it is unknown whether A20 inhibits TRAF6 in rainbow trout using a similar mechanism. To investigate the mechanism of OmA20-mediated TRAF6 regulation, we looked at TRAF6 protein-protein interactions in RTH-149 cells stimulated with LPS using co-immunoprecipitation. Results shown in Figure 6A suggest that OmA20 is indeed physically associated with TRAF6 in RTH-149 cells co-transfected with His-OmA20 and Flag-TRAF6. Interestingly, A20 is known to not only deubiquitinate substrates modified by K63-linked polyubiquitins but can also induce their K48-linked polyubiquitination and degradation via its E3 activity [41]. For example, A20 can deubiquitinate RIP1 via its OTU domain, and it can also act as an E3 ligase to add K48-linked polyubiquitin chains to RIP1 via the ZnF domains, promoting its proteasomal degradation [21]. Therefore, we investigated whether OmA20 might promote K48-linked ubiquitination and proteasomal degradation of TRAF6 in LPS-stimulated RTH-149 cells by examining cell lysates of the OmA20-overexpressed and control (EGFP) cells for the presence of endogenous TRAF6 protein. The stability of endogenous TRAF6 was similar in control and OmA20-overexpressed RTH-149 cells treated with LPS and the proteasome inhibitor MG132 (Figure 6B). We also examined whether the ubiquitination of the TRAF6 was mediated exclusively by K48- or K63-specific linkages in LPS-stimulated RTH-149 cells. Indeed, LPS treatment promoted ubiquitination of TRAF6 with K63-linked chains, but not K48-linked chains, and OmA20 efficiently inhibited LPS-induced TRAF6 ubiquitination in RTH-149 cells with or without MG132 treatment (Figure 6C). These results were further validated by co-expressing various HA-tagged ubiquitin mutants, denoted as K48R in which Lys48 was mutated to Arg, K63R in which Lys63 was mutated to Arg, K48O (K48 Only) in which all lysine residues except Lys48 were mutated to Arg, and K63O (K63 Only) in which all lysine residues except Lys63 were mutated to Arg, together with Flag-TRAF6 in RTH-149 cells, followed by a ubiquitination assay. Here, again, we found increased levels of ubiquitinated TRAF6 in RTH-149 cells overexpressing K48R or K63O ubiquitin compared to those transfected with a K63R or K48O ubiquitin mutants. In addition, K48R or K63O ubiquitin-mediated polyubiquitination of TRAF6 was attenuated by overexpressed OmA20 (Figure S3).

**Figure 7:**
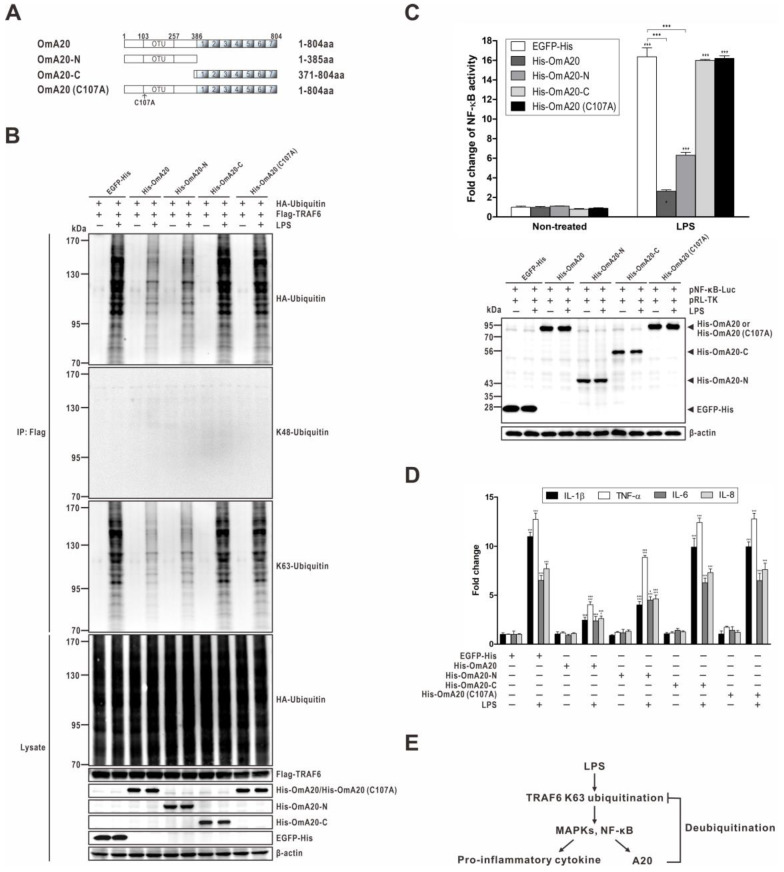
Effect of OmA20 mutants on the ubiquitination of TRAF6 and activation of NF-κB in RTH-149 cells stimulated with LPS. (**A**) A schematic diagram of OmA20 and its mutants used in this study. (**B**) RTH-149 cells were transfected with expression vectors for various combinations (above lanes) of His-OmA20, His-OmA20-N, His-OmA20-C, His-OmA20 (C107A), EGFP-His, Flag-TRAF6, and HA-Ubiquitin. After transfection, cells were stimulated with LPS (10 μg/mL) for 12 h. Flag-TRAF6 was isolated by IP followed by IB (with anti-HA, anti-K48-linkage specific polyubiquitin, and anti-K63-linkage specific polyubiquitin antibodies) to examine its ubiquitin conjugation. Cell lysates were subjected to IB to detect the indicated proteins. All data are representative of three or more experiments. (**C**) RTH-149 cells, co-transfected with His-OmA20, His-OmA20-N, His-OmA20-C, His-OmA20 (C107A), or EGFP-His along with pNF-κB-Luc and pRL-TK for 48 h, were stimulated with LPS (10 μg/mL) for 12 h. Then, NF-κB activity was determined using a dual-luciferase reporter assay system (upper panel). A similar level of overexpression of EGFP, OmA20, and OmA20 mutants was confirmed by western blot analysis using anti-6×His tag antibody (lower panel). (**D**) RTH-149 cells, transfected with His-OmA20, His-OmA20-N, His-OmA20-C, His-OmA20 (C107A), or EGFP-His for 48 h, were stimulated with LPS (10 μg/mL) for 12 h. Then, the expression of IL-1β, TNF-α, IL-6, and IL-8 was analyzed by qRT-PCR. (**E**) A schematic diagram of the regulation of LPS-induced inflammation by OmA20 in RTH-149 cells. The diagram is based on our findings from this study. * *p* < 0.05, *** *p* < 0.001 unstimulated control vs. LPS-stimulated cells; ^●^ *p* < 0.05, ^●●●^ *p* < 0.001 EGFP-His vs. His-OmA20 or His-OmA20 mutants.

The DUB activity of mammalian A20 towards key NF-κB signaling proteins, such as TRAF6, is conferred by the N-terminal OTU domain. Therefore, to determine the physiological significance of the OmA20 OTU domain in regulating the activity of TRAF6 by removing K63-linked chains, we examined the influence of overexpression of OmA20 mutants on the ubiquitination of TRAF6 in LPS-stimulated RTH-149 cells. Wild-type OmA20 and C-terminal ZnF domain deletion mutant OmA20-N disassembled K63-linked ubiquitin chains on TRAF6. In contrast, DUB-deficient mutants, the N-terminal OTU domain deletion mutant OmA20-C and a catalytically inactive DUB mutant OmA20 (C107A), markedly attenuated deubiquitination of K63-linked ubiquitin chains on TRAF6, implying that the OTU domain is required for DUB activity (Figure 7B). TRAF6 is an E3 ligase that primarily generates K63-linked polyubiquitin chains and is involved in LPS-induced NF-κB activation [39,40]. To determine whether OmA20 OTU participates explicitly in suppressing the LPS-mediated NF-κB pathway, we investigated the inhibitory effects of OmA20 and its mutants in RTH-149 cells. Overexpression of OmA20 and OmA20-N, but not OmA20-C and OmA20 (C107A), suppressed LPS-induced NF-κB activation (Figure 7C, upper panel). The expression level of OmA20 and its mutants did not show a significant difference (Figure 7C, lower panel), which confirmed the fold change of NF-κB activity was due to each protein’s activity. Furthermore, overexpression of OmA20 and OmA20-N drastically reduced the expression of IL-1β, TNF-α, IL-6, and IL-8 in RTH-149 cells stimulated with LPS. In contrast, overexpression of DUB-deficient mutants, OmA20-C and OmA20 (C107A), did not affect the expression of these pro-inflammatory cytokines (Figure 7D). Overall, these results suggest that OmA20, via its conserved OTU domain, cleaves ubiquitinated TRAF6, thereby downregulating LPS-induced NF-κB activity.

## 3. Discussion

Because TLR signals commit organisms to inflammatory responses, they must be strictly regulated to protect hosts from excessive inflammation [42,43,44]. The ubiquitin-editing enzyme A20 plays a critical role in mammals by negatively regulating TLR-mediated inflammatory responses [27,45]. The importance of A20 in preventing excessive inflammation has been demonstrated by extensive in vivo research. For example, Lee et al. reported that A20-deficient mice spontaneously develop severe inflammation, are hypersensitive to LPS, and show premature lethality due to severe multi-organ inflammation and cachexia [46]. Uddin et al. reported that immune responsive gene 1 (IRG1)-induced A20 expression significantly decreased TNF-α production in an LPS-stimulated sepsis mice model [47]. In addition, Turer et al. proved that lethal inflammation in A20-deficient mice could be mainly assigned to TLR signaling because mice doubly defective for A20 and the TLR adaptor protein MyD88 do not demonstrate early mortality or cachexia [38]. Although orthologs of human A20 have recently been identified in various fish species, their role in TLR pathways is still largely unknown. In this study, we demonstrated, using gain- and loss-of-function experiments, that OmA20 acts as a negative regulator of TLR signaling in rainbow trout RTH-149 cells in the same way as it does in mammals. When RTH-149 cells were stimulated with LPS, MAPK phosphorylation, and NF-κB activity were increased 12 and 6 h later, respectively (Figure 4A,B). OmA20 overexpression significantly reduced MAPK phosphorylation and NF-κB activity induced by LPS in RTH-149 cells, resulting in lower expression of pro-inflammatory cytokines (Figure 4C,D and Figure 5A). OmA20 silencing, on the other hand, increased MAPK phosphorylation and pro-inflammatory cytokine expression in LPS-stimulated RTH-149 cells (Figure 4C and Figure 5B).

In mammals, it has been shown that A20 can target TRAF6 and downregulate NF-κB activity. TRAF6 is an E3 ubiquitin ligase that regulates the TLR signaling pathway in response to microbial products and cytokines [40,48]. TRAF6 autoubiquitination is required to activate TAK1 and IKK-mediated IκB degradation, which leads to the activation of MAPKs and NF-κB, which, in turn, regulate the expression of pro-inflammatory cytokine genes [49]. In a previous study, Boone et al. reported that mammalian A20 is induced by LPS in macrophages and removes ubiquitin chains from TRAF6, thereby halting TLR-driven NF-κB activity [18]. Mammalian A20 has also been shown to inhibit ubiquitin chain synthesis through disrupting ubiquitin enzyme complexes. Shembade et al. observed that mammalian A20 inhibits the E3 ligase activity of TRAF6 by antagonizing interactions with the E2 enzymes, such as Ubc13 and UbcH5c [26]. Whether A20 limits substrate ubiquitination by cleaving polyubiquitin chains or disrupting ubiquitin enzyme complexes, the restriction of the ubiquitination of signaling proteins is an essential mechanism by which A20 regulates immune signals [50]. We previously demonstrated that TRAF6 might function like those of mammals as a molecular bridge, linking upstream TLRs with the downstream NF-κB and MAPK signaling pathways in rainbow trout [51]. Here, we showed that OmA20 was associated with TRAF6 (Figure 6A) and downregulated K63-linked polyubiquitination of TRAF6 induced by LPS stimulation in RTH-149 cells (Figure 6C and Figure 7B). Moreover, overexpression of DUB-deficient mutants, the N-terminal OTU domain deletion mutant OmA20-C and a catalytically inactive DUB mutant OmA20 (C107A), markedly attenuated deubiquitination of K63-linked ubiquitin chains on TRAF6, indicating that the OTU domain is required for DUB activity (Figure 7B). We also checked the effect of OmA20 mutants on the NF-κB activity and the expression of pro-inflammatory cytokines. Consistently, overexpression of DUB-deficient mutants, OmA20-C and OmA20 (C107A), did not affect the NF-κB activity and the expression of these pro-inflammatory cytokines (Figure 7C,D). Though additional studies are needed to determine the exact mechanism, our findings suggest that OmA20 might function through its conserved OTU domain to cleave ubiquitinated TRAF6, thereby downregulating LPS-induced NF-κB activity (Figure 7E).

## 4. Materials and Methods

### 4.1. Cell Line and Reagents

The rainbow trout hepatoma cell line RTH-149 was obtained from the American Type Culture Collection (ATCC CRL-1710; Rockville, MD, USA) and cultured as previously described [33]. *Escherichia coli* 0111:B4 LPS and MG132 were purchased from InvivoGen (San Diego, CA, USA) and Sigma-Aldrich (St. Louis, MO, USA), respectively. The antibodies against JNK, phosphorylated JNK (Thr183/Tyr185), TAK1, phosphorylated TAK1 (Thr184/187), phosphorylated p38 MAPK (Thr180/Tyr182), K48-linkage specific polyubiquitin, K63-linkage specific polyubiquitin, HA-tag, and β-actin were purchased from Cell Signaling Technology (Beverly, MA, USA). The antibodies against p38 MAPK (ab170099) and TRAF6 (ab33915) were from Abcam (Cambridge, UK). The antibodies against 6×His tag and Flag-tag were from Invitrogen (Carlsbad, CA, USA).

### 4.2. cDNA Cloning of the Gene Encoding OmA20

A first-strand cDNA was synthesized from 1 μg of total RNA isolated from rainbow trout fingerlings (10 g each, provided from a fish farm in Geochang, Korea) as previously described [52]. Based on the EST sequence of a partial A20 (GenBank Accession number DQ400414.1), two intragenic primers (A20-IGP-R and A20-IGP-F for 5′- and 3′-RACE, respectively) were designed and used as gene-specific primers for amplifying the 5′- and 3′-ends of OmA20 cDNA. The 5′- and 3′-RACE products were cloned into a pGEM-T easy vector (Promega, Madison, WI, USA), sequenced, and assembled. Finally, the full-length cDNA was verified by amplifying an ORF using a pair of primers (A20FL-F/A20FL-R). All the primers used in this study were listed in Table S1.

### 4.3. Sequence Analysis

The deduced amino acid sequence of OmA20 was analyzed for similarity using the BLAST program at the ExPASy Molecular Biology Server (http://www.expasy.org/tools/blast/; last accessed date: 7 September 2021). An OTU domain and seven ZnF domains of OmA20 were predicted by the Simple Modular Architecture Research Tool (SMART) and Pfam servers (http://smart.embl-heidelberg.de/ and http://pfam.xfam.org/, respectively; last accessed date: 7 September 2021). A multiple sequence alignment analysis was performed using the MegAlign program of the DNASTAR Lasergene software package (Madison, WI, USA). A phylogenetic tree analysis was carried out using the Neighbor-Joining (NJ) method with 10,000 bootstrap replicates in the MEGA-X software (Pennsylvania State University, University Park, PA, USA). Accession numbers of proteins used in the sequence analysis were listed in Table S2.

### 4.4. RNA Extraction, cDNA Synthesis, and Quantitative Real-Time PCR

Total RNA was extracted using the RNeasy plus mini kit (Qiagen, Hilden, Germany) from tissue samples and RTH-149 cells: eight different tissues (eye, gill, head kidney, intestine, kidney, liver, skin, and spleen) were obtained from four fish separately; RTH-149 cells (1 × 10^6^ cells/well in 6-well plates) were stimulated with different concentrations of LPS for the indicated time periods. Reverse transcription of 1 μg total RNA from each sample was performed using an iScript cDNA synthesis kit (Bio-Rad Laboratories, Inc., Hercules, CA, USA) following the manufacturer’s instructions. qRT-PCR was performed using iQ^TM^ SYBR^®^ Green Supermix (Bio-Rad Laboratories, Inc.) as previously described [52].

### 4.5. Western Blot Analysis

RTH-149 cells (1 × 10^6^ cells/well in 6-well plates) were stimulated with LPS (10 μg/mL) for the indicated time periods. Cells were harvested and lysed in RIPA buffer (Bio-Rad Laboratories, Inc.) containing protease and phosphatase inhibitors on ice for 30 min. Equal amounts of denatured protein samples (40 μg) were loaded onto 12% SDS-PAGE. Then, the gels were transferred to polyvinyl difluoride membranes (Bio-Rad Laboratories, Inc.), the transferred membranes were blocked with 5% (*w/v*) BSA in TBS containing 0.1% Tween-20 for 1 h, and immunoblotting was carried out with appropriate antibodies. Chemiluminescence was detected using an ECL kit (Bio-Rad Laboratories, Inc.) following the manufacturer’s protocol.

### 4.6. Construction of Expression Plasmids

ORFs of OmA20 and EGFP were amplified from OmA20 full-length cDNA clone and pEGFP-N2 plasmid (Clontech Laboratories, Inc., Mountain View, CA, USA), respectively, by PCR with the gene-specific primer sets (A20 PE-F/A20 PE-R for OmA20; EGFP PE-F/EGFP PE-R for EGFP). OmA20 deletion mutants (OmA20-N and OmA20-C) were generated by standard PCR methods with each primer set (A20 PE-F/A20-N PE-R for OmA20-N; A20-C PE-F/A20 PE-R for OmA20-C). To introduce a mutation at the Cys residue in the OTU domain of OmA20, which is essential for DUB function [53], OmA20 (C107A) was generated by overlap extension PCR with two sets of primers [A20 PE-F/A20 (C107A) PE-R and A20 (C107A) PE-F/A20 PE-R] as described previously [54]. In brief, two fragments of DNA, amplified in the first round of PCR with each primer set, were combined and amplified again by the second round of PCR with the gene-specific primer set (A20 PE-F/A20 PE-R). ORFs of TRAF6, ubiquitin, ubiquitin (K48R), ubiquitin (K63R), ubiquitin (K48O), and ubiquitin (K63O) were amplified from rainbow trout first-strand cDNA, pRK5-HA-Ubiquitin-WT Addgene plasmid #17608, pRK5-HA-Ubiquitin-K48R Addgene plasmid #17604, pET3a-Ub-K63R Addgene plasmid #18898, pRK5-HA-Ubiquitin-K48 Addgene plasmid #17605, and pRK5-HA-Ubiquitin-K63 Addgene plasmid #17606 (Addgene Inc., Watertown, MA, USA), respectively, by PCR with the gene-specific primer sets [TRAF6 PE-F/TRAF6 PE-R for TRAF6; Ubiquitin PE-F/Ubiquitin PE-R for ubiquitin; Ubiquitin (K48R/K63R) PE-F/Ubiquitin PE-R for ubiquitin (K48R) and ubiquitin (K63R); Ubiquitin (K48O/K63O) PE-F/Ubiquitin PE-R for ubiquitin (K48O) and ubiquitin (K63O)]. The PCR products were digested with *Hind*III and *Xho*I (New England Biolabs, Ipswich, MA, USA) before being subcloned into pcDNA4.1 (Invitrogen) digested with the same restriction enzymes. The resultant plasmids were named His-OmA20, EGFP-His, His-OmA20-N, His-OmA20-C, His-OmA20 (C107A), Flag-TRAF6, HA-Ubiquitin, HA-Ubiquitin (K48R), HA-Ubiquitin (K63R), HA-Ubiquitin (K48O), and HA-Ubiquitin (K63O).

### 4.7. Overexpression of OmA20 and OmA20 Mutants in RTH-149 Cells

RTH-149 cells (1 × 10^6^ cells/well in 6-well plates) were transfected with His-OmA20, His-OmA20-N, His-OmA20-C, His-OmA20 (C107A), or EGFP-His (control) using the Lipofectamine 2000 (Invitrogen). Overexpression of OmA20, OmA20 mutants, and EGFP was confirmed at the protein level by western blot analysis using a 6×His tag antibody (Invitrogen). At 48 h after transfection, cells (except control cells) were stimulated with LPS (10 μg/mL) for 12 h and collected to assess targets at the protein and gene expression level by western blot and qRT-PCR, respectively, as described above.

### 4.8. Silencing of OmA20 Expression in RTH-149 Cells

OmA20 siRNA gene silencing was performed, as previously described [33]. In brief, RTH-149 cells (1 × 10^5^ cells/well in 6-well plates) were transfected with 24 nM siRNA against OmA20 or control siRNA for 48 h using the Lipofectamine 2000 (Invitrogen). Then, cells (except control cells) were stimulated with LPS (10 μg/mL) for 12 h and collected to assess targets at the protein and gene expression level by western blot and qRT-PCR, respectively, as described above. siRNA sequences used in this study were as follows: OmA20, 5′-CGU UAC AGC ACC AUG AAU UGG UU-3′ (sense) and 5′-CCA AUU CAU GGU GCU GUA ACG UU-3′ (antisense) and control, 5′-AGA UCC GCU ACU GUC CGA AUU-3′ (sense) and 5′-UUC GGA CAG UAG CGG AUC UUU-3′ (antisense).

### 4.9. Dual-Luciferase Activity Assay

After co-transfecting RTH-149 cells with luciferase reporter plasmids pNF-κB-Luc (Clontech Laboratories, Inc.) and pRL-TK (internal control; Promega) for 48 h, cells (except control cells) were stimulated with LPS (10 μg/mL) for the indicated time periods. Then, cells were lysed with lysis buffer (Promega), and firefly and *Renilla* luciferase activities were measured using the Dual-Luciferase Assay System (Promega). Firefly luciferase activity was normalized to that of *Renilla*. In some experiments, cells were co-transfected with His-OmA20, His-OmA20-N, His-OmA20-C, His-OmA20 (C107A), or EGFP-His along with pNF-κB-Luc and pRL-TK, in order to examine the effect of OmA20 and OmA20 mutants on the activation state of NF-κB.

### 4.10. Immunoprecipitation

Immunoprecipitation assays were performed using a Pierce Direct Magnetic IP/Co-IP kit (Rockford, IL, USA) following the manufacturer’s protocol. Briefly, RTH-149 cells were co-transfected with His-OmA20 and Flag-TRAF6 for 48 h. Then, cells were stimulated with LPS (10 μg/mL) for 12 h, lysed with IP lysis buffer, and protein levels were quantified. Five micrograms of 6×His tag or Flag tag antibodies were coupled to the *N*-hydroxysuccinimide-activated magnetic beads in an amine-free buffer. Then, 500 μL of cell lysates (2 mg/mL) were subjected to immunoprecipitation by incubation (overnight at 4 °C) with the prepared antibody-coupled beads. After extensive washing, the beads were spun down, resuspended, and boiled. Pull-down samples were detected using western blotting as described above.

### 4.11. Ubiquitination Assay

RTH-149 cells were transfected for 48 h with Flag-TRAF6 and various constructs as indicated. Then, cells (except control cells) were stimulated with 10 μg/mL LPS for 12 h and next treated with or without MG132 (25 μM) for an additional 4 h to block proteasomal degradation. Cell lysates were collected and immunoprecipitated with Flag-tag antibody (5 μg). Pull-down samples were subjected to immunoblotting with K48-linkage specific polyubiquitin, K63-linkage specific polyubiquitin, and HA-tag antibodies to visualize polyubiquitinated TRAF6 protein bands.

### 4.12. Statistical Analysis

GraphPad Prism software (version 5, GraphPad Software Inc., San Diego, CA, USA) was used for graphing and statistical analysis. One-way ANOVA followed by Tukey’s post hoc test was applied to multiple-group comparisons. All experiments were performed in triplicate, repeated at least three times, and the results were expressed as mean ± SEM. The statistical significance was accepted as *p* < 0.05.

## 5. Conclusions

In this study, we have identified and characterized A20 in rainbow trout, *O. mykiss*. Gain- and loss-of-function experiments showed that OmA20 inhibits MAPK and NF-κB activation, as well as the expression of pro-inflammatory cytokines in RTH-149 cells stimulated with LPS. Mechanistically, OmA20 interacted directly with TRAF6 and deubiquitinated LPS-induced K63-linked polyubiquitination of TRAF6 in a DUB activity-dependent manner. Although additional in vivo studies are needed to clarify the current in vitro data, our findings indicate that OmA20 reduces LPS-induced inflammation in rainbow trout, most likely by regulating K63-linked ubiquitination of TRAF6. The knowledge of OmA20 as a negative regulator of the TLR signaling pathway will help to provide insights into the development of new control strategies for the inflammatory diseases of fish.

## Data Availability

The data presented in this study are available within the Supplementary Material.

## Supplementary Materials

The following are available online at https://www.mdpi.com/article/10.3390/ijms22189801/s1.
