# Supplementary material for: A20 Inhibits LPS-Induced Inflammation by Regulating TRAF6 Polyubiquitination in Rainbow Trout
Source: Int J Mol Sci. 2021 Sep 10;22(18):9801. doi: 10.3390/ijms22189801 (PMC8472768; doi:10.3390/ijms22189801)
Supplement: Supplementary file 1 [file ijms-22-09801-s001.zip › ijms-1366766-supplementary.pdf]

Supplementary Information for:

A20 Inhibits LPS-Induced Inflammation by Regulating TRAF6 Polyubiquitination in Rainbow Trout

Ju Hye Jang[1], Hyun Kim[1], In Young Jung[2], Ju Hyun Cho[1,2,3,*]

[1]Research Institute of Life Sciences, Gyeongsang National University, Jinju 52828, South Korea

[2]Division of Applied Life Science (BK21Four), Gyeongsang National University, Jinju 52828, South Korea

[3]Division of Life Science, Gyeongsang National University, Jinju 52828, South Korea

[*]Corresponding author:

Ju Hyun Cho

Division of Life Science, Gyeongsang National University, 501 Jinju Dae-ro, Jinju 52828, South Korea

Phone: 82-55-772-1347. Fax: 82-55-772-1349. E-mail address: juhyun.cho@gnu.ac.kr

Includes 3 figures and 2 tables.

```
   1  GGG GGA TAG GGG AGA AAG CTG AAT GGC GTT GTG TGG ACT GCT GCG GTT AAC CTG ATT GAA GTG GCA GAG GAC TAC CCG GAG CAT CCT TAG   90
  91  GCT TTG TTG GTA GTC TTT GTA CCG ACA TAT TGC ACA GTT CTG CGT TTG TAC ACA TTA CAT CTG GTG AAC ACC ATG TCC CAG GGA CAG AAT  180
                                                                                                  M   S   Q   G   Q   N

 181  TTC CTG CCC AAG TTC CTC TTT GTG AGT AAC CTG CTG AAA GCG GTC AAG ATC CGG GAG CGG GTG CCC AAC GAT GTG GTG AAG CCG GCG GCC  270
       F   L   P   K   F   L   F   V   S   N   L   L   K   A   V   K   I   R   E   R   V   P   N   D   V   V   K   P   A   A

 271  AGC GGA GGC CTG ATG CAC CAC CTG CGG GGC ATG CAC CGC TAC ACC CTG GAG ATG ATC CGC ATG AGC CAG TTC CCC CAG GCC TTC CAA CAG  360
       S   G   G   L   M   H   H   L   R   G   M   H   R   Y   T   L   E   M   I   R   M   S   Q   F   P   Q   A   F   Q   Q

 361  GTC ATC CAG GCA GCC ATC TTG GAC AGG GCC ATG CAG AGC TCC CTA GAG CAG GAG AAG AAG CTC AAC TGG TGC CGC GAG GTC AAG AAG ATG  450
       V   I   Q   A   A   I   L   D   R   A   M   Q   S   S   L   E   Q   E   K   K   L   N   W   C   R   E   V   K   K   M

 451  GTG CCA TTG AGA ACC AAT GGA GAT GGG AAC TGT CTT CTC CAT GCT GCA TCT CAG TAC ATG CTG GGG GTG CAG GAC ACA GAC CTT GTC CTG  540
       V   P   L   R   T   N   G   D   G   N   C   L   L   H   A   A   S   Q   Y   M   L   G   Q   V   D   T   D   L   V   L

 541  AGG AAG GCC CTG TTC AGC GTG CTA AAG GAG ACA GAC ACG GGC AAC TTC AGA GCC CGT TTC CAG ACT GAG CTG CTG CAG TCT CAG GAG TTC  630
       R   K   A   L   F   S   V   L   K   E   T   D   T   G   N   F   R   A   R   F   Q   T   E   L   L   Q   S   Q   E   F

 631  ACC CAG ACT GGC CTG CGT TAC AGC ACC ATG AAT TGG GAG GAG GAA TGG GAG AAG ATC ATC AAG ATG GCA TCA CCT GTA TCC AGC AGC AAC  720
       T   Q   T   G   L   R   Y   S   T   M   N   W   E   E   E   W   E   K   I   I   K   M   A   S   P   V   S   S   S   N

 721  GGG CTG CAG TTT GAC TCC CTG GAA GAC ATA CAC ATC TTT GTC CTC TCA AAC ATC CTC CGC AGG CCC ATC ATC GTC ATC GCA GAT CAG GTG  810
       G   L   Q   F   D   S   L   E   D   I   H   I   F   V   L   S   N   I   L   R   R   P   I   I   V   I   A   D   Q   V

 811  CTA CGT AGC ATG AAA TCC TGC TCC TCC TTC TCT CCC CTC AAT GTG GGG GGC ATC TAC CTG CCT TTA CAC TGG CCT CCA GGG GAG TGC TAC  900
       L   R   S   M   K   S   C   S   S   F   S   P   L   N   V   G   G   I   Y   L   P   L   H   W   P   P   G   E   C   Y

 901  AAG TAC CCC ATA GTG CTC GGC TAC GAC TCC CAG CAC TTT GCC CCC CTC ATC ACC ATC AAA GAC AGT GGC CCG GAG ATC CGA GCG GTG CCG  990
       K   Y   P   I   V   L   G   Y   D   S   Q   H   F   A   P   L   I   T   I   K   D   S   G   P   E   I   R   A   V   P

 991  CTG ATC AAC CCG GGT CGT GGG GAC TTC GAG GAG CTC AAG GTG CAC TTT CTG ATG GAG AAG GAG CAG ACG CAG AAG GAG AGG CTG CTG AAG 1080
       L   I   N   P   G   R   G   D   F   E   E   L   K   V   H   F   L   M   E   K   E   Q   T   Q   K   E   R   L   L   K

1081  GAC TAC CTA ATG CTG ATT GAG ATC CCT GTC ATT GGA CTG GGC TAC GAT ACC ACA CGC ATC ATC AAC GCT GCC AGG CTG GAT GAA GGG AAC 1170
       D   Y   L   M   L   I   E   I   P   V   I   G   L   G   Y   D   T   T   R   I   I   N   A   A   R   L   D   E   G   N

1171  CTG CCT GAG GAC ATG AAC CTG ATG GAG GAC TAC CTC CAG TTG GTC AAC CAC GAG TAC AAG CGC TGG CAG GAG GAC AAG GAC CAA CTG TGG 1260
       L   P   E   D   M   N   L   M   E   D   Y   L   Q   L   V   N   H   E   Y   K   R   W   Q   E   D   K   D   Q   L   W

1261  GCC GCC CCC ACC CCC CAT AGA CCA CCC CCC TTC TCC GTG TCT CAG CTG TCC CTC ATC GAG ATC CGA TGT GCC ACA CCA CGG TGC ACC TTC 1350
       A   A   P   T   P   H   R   P   P   P   F   S   V   S   Q   L   S   L   I   E   I   R   C   A   T   P   R   C   T   F

1351  TAC GTC TCA GTG GAC ACC CAG CCA CAC TGC CAC GAG TGC TTT GAG AAA CGC CAG GGC CAG CCT AAC GGG AGG ATA GAG GCT GCC TCT ACG 1440
       Y   V   S   V   D   T   Q   P   H   C   H   E   C   F   E   K   R   Q   G   Q   P   N   G   R   I   E   A   A   S   T

1441  AAA GGC GGA AAT CAG GCT TGT GAG CCG GGG TGT AGT AGT AGA GTA CGG CCC AGC CCC TCT GGC TGC CGG GCG GTG TTA TCC AGC CCG CGC 1530
       K   G   G   N   Q   A   C   E   P   G   C   S   S   R   V   R   P   S   P   S   G   C   R   A   V   L   S   S   P   R

1531  TCC GTC CCT CCA CCG CCC ACG GCG TCC TGC TGT TAC AGC GAA ACC CAC GCC ATG AAG TGC AAG ACA CCC GGC TGC CTT TTC ACC CTC AGC 1620
       S   V   P   P   P   P   T   A   S   C   C   Y   S   E   T   H   A   M   K   C   K   T   P   G   C   L   F   T   L   S

1621  GTG GAG CAC GAT GGT CTG TGT GAA CGC TGC TTC AAC ACC CGG CAG AAT GCA CCC ACT GCT GAT GGC CCC TCG GGC CTC CCC CTG CCT GCC 1710
       V   E   H   D   G   L   C   E   R   C   F   N   T   R   Q   N   A   P   T   A   D   G   P   S   G   L   P   L   P   A

1711  CAC CTC GGC TGG CCC CAG TGG GGC GCG GGA CGG GAG CAA GAG ACA GAG CGG TGC AAC GTG TGC CAC CAG AAG GCC TTC AGG ATA TTT AAC 1800
       H   L   G   W   P   Q   W   G   A   G   R   E   Q   E   T   E   R   C   N   V   C   H   Q   K   A   F   R   I   F   N

1801  GGA CTG TGC CCC CCG TGC CTC CAG AGG ACT GCT ACG GAG AGG GGC GAG GAT CCC CAG CCG CCG GCA ACA GCC AGG ATA GAG GCC TCC TCT 1890
       G   L   C   P   P   C   L   Q   R   T   A   T   E   R   G   E   D   P   Q   P   P   A   T   A   R   I   E   A   S   S

1891  GGG TCG TTA TGG ACC CAG CCC CAG CCA AGG GAC TCT GAG CGC TCA GGC ACC TCG GCT CTC AAC GGC CAC ACG TCT GGC CGG CCC TGT AAG 1980
       G   S   L   W   T   Q   P   Q   P   R   D   S   E   R   S   G   T   S   A   L   N   G   H   T   S   G   R   P   C   K

1981  AGG TCG GGA TGC CAG TTC TTC GGG ACT CAA CAG AAT CTT GGA TTC TGC ACC ATC TGT TAC CTG GAC TAT CAG ACC AAC CAC CTG GCG ACC 2070
       R   S   G   C   Q   F   F   G   T   Q   Q   N   L   G   F   C   T   I   C   Y   L   D   Y   Q   T   N   H   L   A   T

2071  CCT CAT CCT CCC CCT GCT CCA ATC CAG AGT CGT CAC GCG TCA GAG GCA GGT TTC CAG GAT GCT TCG CGC TGT CGG GGC CCG GAC TGT GGG 2160
       P   H   P   P   P   A   P   I   Q   S   R   H   A   S   E   A   G   F   Q   D   A   S   R   C   R   G   P   D   C   G

2161  GCC ATG GGC AAG GTC ATG CTG GAG GGT TAC TGC AAC AAG TGC TAC GTA AAG GAG CAG AGC ACC AGG CTC AAC CAG GCT GCT ACC AGG GCT 2250
       A   M   G   K   V   M   L   E   G   Y   C   N   K   C   Y   V   K   E   Q   S   T   R   L   N   Q   A   A   T   R   A

2251  CCC GGC ACC CAC TCC CCT CCA CTG GTC ATG CGA ACA GCT AAA CCA GAA AGA GAC AGA ACC AGA CAG ACG CAG ACC CAG ACC CAG ACC CAG 2340
       P   G   T   H   S   P   P   L   V   M   R   T   A   K   P   E   R   D   R   T   R   Q   T   Q   T   Q   T   Q   T   Q

2341  GCA ACG TGT AGG CGG AGT GGC TGC AGT AAC ATC TCC CCC GGC TGC ACA GAC CTG TGT CCA GAG TGC CAC ACG CGA AAC ACG CGG GGG CAG 2430
       A   T   C   R   R   S   G   C   S   N   I   S   P   G   C   T   D   L   C   P   E   C   H   T   R   N   T   R   G   Q

2431  AGA GAG AGT CGC AGG GAG AGG GCA GGG GCA CCT AAA GAG AAG ACA AAG CAG CGC TGT AAA ACC CTA GGC TGT GAG CAC TAT GCT AAC CAA 2520
       R   E   S   R   R   E   R   A   G   A   P   K   E   K   T   K   Q   R   C   K   T   L   G   C   E   H   Y   A   N   Q

2521  GAG AAG CAA GGC TAC TGT AAT GAA TGT GAC CTC TTT AAG CAG ATC TAC AGG GGG TGA GCC CAA AGG TTT GGG AGG ATT AAA ATG GCC GTG 2610
       E   K   Q   G   Y   C   N   E   C   D   L   F   K   Q   I   Y   R   G   *

2611  CCA CGA TGC TAG TCA ATG CAT CTC TGA AAC TAG TGA CCT TAC GGC CAA AAG CCC ATT TTA AAA TGT GCT CAT GAA GAG CGG GGG CAG TAG 2700
2701  AAA TGG CCG GCA GAA TAC CTC TTA GTG CAA TTA TTA TTA TCT CAC GTG TAC AGG ACG AGA GGT GAG GTA TAC GTG TCA TAA AAC TGA CAA 2790
2791  CAT ATG TAC AGT ATG TAT CTA AGT ACA TAC TGT ATC ATG CTG GAC TAC TGA TTG TGT CTT GAA AGA AGC GCA ATG TGT AAA CTG CTT AAT 2880
2881  AAT ACG GCC AAG GAA TGA TCT ATC AAG CAG CTA GCT ACC TAC ACA ATA ATG TGA TAA ACC ACA GCT TTT GTT TAA GAA CCT CAT TGA CCA 2970
2971  AAG TTC TGA CAA GTG CAG AGC TGT GCC AAA GCC ATC ATG CAC TGC CAA AAG GAG TTT GTT TAG TCA AAA TTC GGT TTT AAA CAG GAG TAG 3060
3061  TTA CAG TCT CAA AAA CTG CAC CTA CCT AGA CAG CTA ATG TAC AGT CTC TGA GCA TTT GCG AGT GGG CAG GAC CCT TAA AAG TAT GGT GCC 3150
3151  ACT TGT ACA GAA AGC AAA CAT GGA GGT TCT GTG ACA TGA CAG AGA AAA CAG TAG TTA GAA CTC AAT AAT ATT CAA CCT CTC CCA CCG CCG 3240
3241  CAT AGA TGT CTG CTG GCA GTA CAG ATG CTG TTG CCT TTT GTG GTT GTG TTC TTG TTT GCT GTT GAC CAC TTT TGT ATC TGT ACT GTA GAA 3330
3331  ATA CAA GTT AAT GCT TGC CCT GAA AAA AAA AAA AAA A                                                                         3367
```

Figure S1. Nucleotide and predicted amino acid sequences of OmA20. An OTU and seven ZnF domains are boxed and underlined, respectively. Stop codon is indicated with an asterisk. The cDNA sequence of OmA20 was registered in the GenBank under the accession number MF671983.

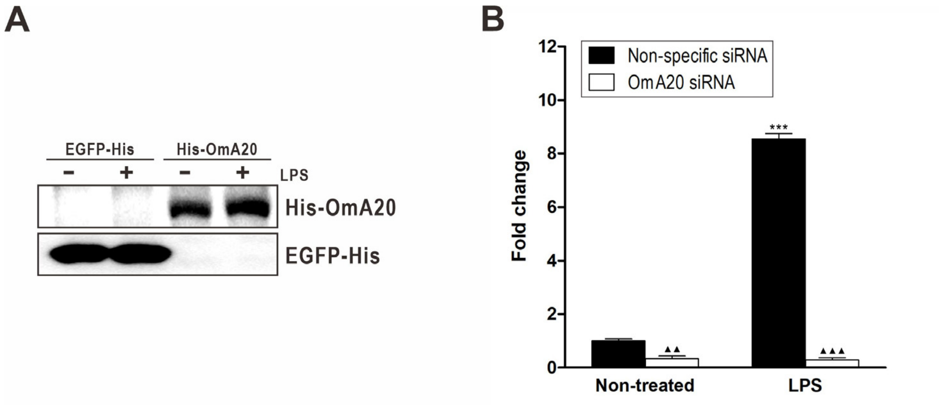

Figure S2. Overexpression and silencing of OmA20 gene in RTH-149 cells. (A) RTH-149 cells, transfected with His-OmA20 or EGFP-His for 48 h, were stimulated with LPS (10 μg/ml) for 12 h. Overexpression of OmA20 and EGFP was confirmed by western blot analysis using anti-6×-His tag antibody. (B) RHT-149 cells, transfected with OmA20 siRNA or non-specific siRNA for 48 h, were stimulated with LPS (10 μg/ml) for 12 h. The expression of OmA20 was analyzed by qRT-PCR. $^{***}P < 0.001$ unstimulated control versus LPS-stimulated cells; ▲▲$P <$ 0.01, ▲▲▲$P < 0.001$ non-specific siRNA versus OmA20 siRNA.

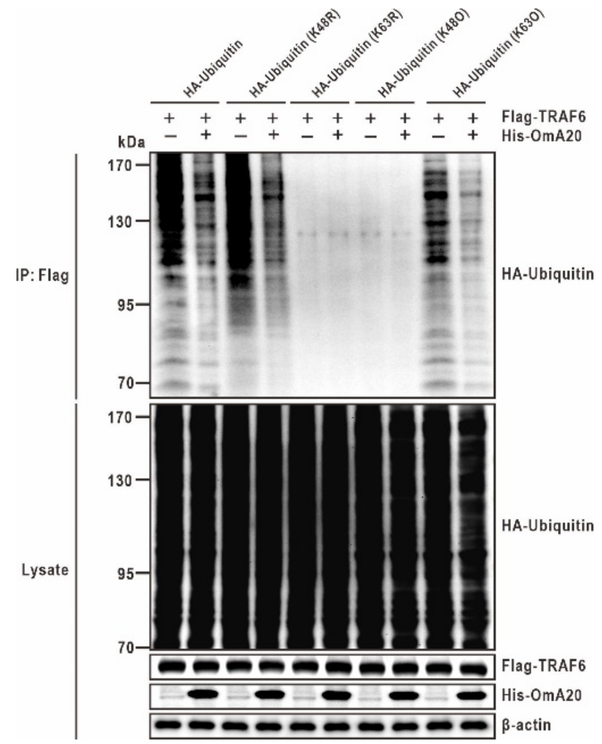

Figure S3. Inhibition of TRAF6 K63-linked ubiquitination by OmA20 overexpression in RTH-149 cells stimulated with LPS. Flag-TRAF6 along with HA-Ubiquitin or HA-Ubiquitin mutants (K48R, K63R, K48O, or K63O) were co-transfected with or without His-OmA20 in RTH-149 cells. After transfection, cells were pretreated with 10 μg/ml LPS for 12 h and treated with MG132 (25 μM) for an additional 4 h. Cell lysates were subjected to IB to detect the indicated proteins. Flag-TRAF6 was isolated by IP followed by IB (with anti-HA antibody) to examine its ubiquitin conjugation. Data shown are representative of three independent experiments.

Table S1. List of primer sequences used in the study.

Name	Sequence	Length (nt)	Usage
A20-IGP-F	5'-CCT CCT TCT CTC CCC TCA ATG TGG-3'	24	3'-RACE
A20-IGP-R	5'-CGT GTC TGT CTC CTT TAG CAC GC-3'	23	5'-RACE
A20FL-F	5'-ATG TCC CAG GGA CAG AAT TTC CTG-3'	24	cDNA cloning
A20FL-R	5'-TCA CCC CCT GTA GAT CTG CTT AAA G-3'	25	cDNA cloning
A20 PE-F	5'-AAG CTT ATG CAT CAT CAC CAT CAC CAT ATG TCC CAG GGA CAG AAT TTC CTG-3'	51	Vector construction
A20 PE-R	5'-CTC GAG TCA CCC CCT GTA GAT CTG CTT AAA GAG-3'	33	
A20-N PE-R	5'-CTC GAG TCA GAT GAG GGA CAG CTG AGA CAC-3'	30	Vector construction
A20-C PE-F	5'-AAG CTT ATG CAT CAT CAC CAT CAC CAT ATG CCC CAT AGA CCA CCC CCC TTC-3'	51	
A20 (C107A) PE-F	5'-GGA GAT GGG AAC GCT CTT CTC CAT G-3'	25	Mutation
A20 (C107A) PE-R	5'-CAT GGA GAA GAG CGT TCC CAT CTC C-3'	25	
EGFP PE-F	5'-AAG CTT ATG GTG AGC AAG GGC GAG GAG C-3'	28	Vector construction
EGFP PE-R	5'-CTC GAG CTT GTA CAG CTC GTC CAT GCC GAG-3'	30	
TRAF6 PE-F	5'-AAG CTT ATG GAC TAC AAA GAC GAT GAC GAC AAG ATG TCC TGC TTT GAG AGT GAT AAG-3'	57	Vector construction
TRAF6 PE-R	5'-CTC GAG TCA CAG CGA GGC CTC GGG CC-3'	26	
Ubiquitin PE-F	5'-AAG CTT ATG TAC CCA TAC GAT GTT CCA GAT TAC GCT ATG CAG ATC TTC GTG AAG ACC-3'	57	Vector construction
Ubiquitin (K48R/K63R) PE-F	5'-AAG CTT ATG TAC CCA TAC GAT GTT CCA GAT TAC GCT ATG CAG ATC TTC GTC AAG ACG-3'	57	Vector construction
Ubiquitin (K48O/K63O) PE-F	5'-AAG CTT ATG TAC CCA TAC GAT GTT CCA GAT TAC GCT ATG CAG ATC TTC GTC AGA ACG-3'	57	Vector construction
Ubiquitin PE-R	5'-CTC GAG TCA ACC ACC TCT GAG ACG GAG-3'	27	
A20F	5'-TGT TCA GCG TGC TAA AGG AGA CAG-3'	24	qRT-PCR
A20R	5'-ATC TGC GAT GAC GAT GAT GGG C-3'	22	
IL-1βF	5'-ACA TTG CCA ACC TCA TCA TCG-3'	21	qRT-PCR
IL-1βR	5'-TTG AGC AGG TCC TTG TCC TTG-3'	21	
IL-6F	5'-CCT TGC GGA ACC AAC AGT TTG-3'	21	qRT-PCR
IL-6R	5'-CCT CAG CAA CCT TCA TAT GGT C-3'	22	
IL-8F	5'-AGA ATG TCA GCC AGC CTT GT-3'	20	qRT-PCR
IL-8R	5'-TCT CAG ACT CAT CCC CTC AGT-3'	21	
TNF-αF	5'-GGG GAC AAA CTG TGG ACT GA-3'	20	qRT-PCR
TNF-αR	5'-GAA GTT CTT GCC CTG CTC TG-3'	20	
EF1-αF	5'-GCT GGA CAA GCT GAA GGC TGA G-3'	22	qRT-PCR
EF1-αR	5'-AGC GCA ATC AGC CTG AGA GGT A-3'	22	

*Hind*III and *Xho*I restriction sites are underlined, and the His, Flag and HA sequences are double-underlined in the primers.

Table S2. A20 protein sequences used for multiple sequence alignment and phylogenetic tree construction.

Species	Accession no.	Species	Accession no.
Gouania willdenowi	XP_028324515.1	*Pangasianodon hypophthalmus*	XP_026795215.1
Maylandia zebra	XP_004551477.2	*Ictalurus punctatus*	XP_017320207.1
Xiphophorus couchianus	XP_027863189.1	*Anas platyrhynchos*	XP_027310259.1
Perca flavescens	XP_010741022.1	*Gallus gallus*	XP_015139672.1
Larimichthys crocea	XP_019951045.1	*Bos taurus*	NP_001179099
Paralichthys olivaceus	XP_026210285.1	*Ovis aries*	XP_004011421
Takifugu rubripes	BUA61356	*Lagenorhynchus obliquidens*	XP_026941172.1
Esox lucius	XP_019902489.1	*Sus scrofa*	XP_005659239.1
Salvelinus malma	AYG86901.1	*Cricetulus griseus*	ERE84112.1
Oncorhynchus mykiss	MF671983	*Mus musculus*	NP_033423
Salmo salar	SPC69637.1	*Rattus norvegicus*	XP_006222897.1
Danio rerio	XP_692922.3	*Homo sapiens*	ACN87240.1
Pimephales promelas	SPC69639.1	*Piliocolobus tephrosceles*	XP_023046727
Cyprinus carpio	XP_018960661.1	*Canis lupus familiaris*	XP_013967796
Anabarilius grahami	ROJ36054.1	*Acinonyx jubatus*	XP_026912585.1
Labeo rohita	RXN27329.1	*Felis catus*	XP_006932110
Electrophorus electricus	XP_026879377.1	*Branchiostoma belcheri tsingtauense*	AGQ17407.1
Tachysurus fulvidraco	XP_027021648.1		
